# Assessment of thyroid cancer risk in more than 334,000 patients with inflammatory bowel disease: a case-control study and a meta-analysis

**DOI:** 10.1186/s12957-018-1485-4

**Published:** 2018-09-10

**Authors:** Lihong Cao

**Affiliations:** Department of Ear-nose-throat, Tianjin Medical University General Hospital, No. 154, Anshan Road, Heping District, Tianjin, 300052 China

**Keywords:** Crohn’s disease, Immunosuppressant, Inflammatory bowel diseases, Thyroid neoplasms, Ulcerative colitis

## Abstract

**Background:**

Potential risk of thyroid cancer in patients with inflammatory bowel disease has not been well investigated. The aim of the study was to reveal the relationship between history of inflammatory bowel disease and risk of thyroid cancer.

**Methods:**

First, 1392 patients with inflammatory bowel disease and 1392 controls were included in a case-control study. All patients did not receive immunosuppressive therapy. A multivariate logistic regression analysis was adopted to determine the relationship between history of inflammatory bowel disease and risk of thyroid cancer. Second, a literature search was performed and eight articles were collected. Pooled odds ratios with 95% confidence intervals were reported for relevant risk estimates in fixed or random effect model.

**Results:**

In the case-control study, thyroid cancer was more common in patients with inflammatory bowel disease than in controls (*P* = 0.032). After Bonferroni correction, association of thyroid cancer risk with history of total inflammatory bowel disease or its two subtypes was not found. In the meta-analysis, patients with total inflammatory bowel disease or ulcerative colitis showed an increased risk of thyroid cancer, but patients with Crohn’s disease did not. Furthermore, inflammatory bowel disease patients with immunosuppressive therapy showed an increased risk of the cancer, but patients without immunosuppressive therapy did not have this finding.

**Conclusions:**

Risk of thyroid cancer probably elevates in patients with inflammatory bowel disease. Inflammatory bowel disease (particularly ulcerative colitis) itself and use of immunosuppressant might contribute to the development of the cancer.

## Background

Thyroid cancer (TC) is one kind of endocrine tumors, which originates in the tissue of thyroid gland [1]. Papillary thyroid carcinoma (PTC) is the most common histological subtype and accounts for more than 80% of TC cases. It often occurs at the age of 35–65 years old and usually affects women more than men [2]. In recent years, incidence of TC has significantly increased [3]. Presently, there are more than three million TC patients around the world [3]. Though prognosis of TC is not very poor, it still causes tens of thousands of deaths each year. Family history, radiation exposure, iodine intake abnormality, and obesity are major risk factors [4–7]. Other potential risk factors have not been well investigated.

Inflammatory bowel disease (IBD) including ulcerative colitis (UC) and Crohn’s disease (CD) is a precancerous disease of colorectal cancer [8]. Besides, an increased risk of extra-intestinal cancer in IBD patients has been revealed by previous studies [9]. According to these findings, potential relationship between risk of TC and history of IBD has also been explored. Some studies reported that risk of TC was elevated in patients with IBD [10, 11], but other studies failed to do so [12, 13]. Thus, a firmed conclusion has not been drawn.

Therefore, we conducted a case-control study and a meta-analysis to evaluate the relationship between the risk of TC and the history of IBD and to further explore the mechanism involved.

## Methods

### Case-control study

Outpatients in Tianjin Binjiang Hospital between 1991 and 2000 enrolled in a health system database, which contained the huge data of these patients from baseline to 2015. All these outpatients signed the written informed consents and agreed to participate in a series of clinical studies including this study. A total of 1392 IBD patients (1022 UC patients, 370 CD patients) were identified in this database according to ICD-9 codes (codes of CD: 555, 550.0, 555.1 and 555.9; codes of UC: 556, 556.0, 556.1, 556.2, 556.3, 556.5, 556.6, 556.8, and 556.9) and served as the IBD group. All these patients did not have any kind of cancer on admission. And, 1392 age- and gender-matched patients with diverticulitis (codes of diverticulitis 62.11 and 562.13) were randomly collected from the database and served as control group.

Research data such as demographic information, personal history, medical history, treatment history, and other useful information from baseline to 2015 were collected from the database. Major endpoint was the development of PTC, and all the patients with PTC should be confirmed pathologically. Total follow-up period of these IBD patients were 27,448 person-years.

Most of the IBD patients received 5-ASA treatment and other supportive treatment. They did not receive any kind of biologics or immunosuppressant. They did not receive any surgical therapy.

History of smoking was defined as smoking (≥ 7 cigarettes per week) for more than 6 months in one’s life. History of drinking was defined as drinking alcohol (≥ 3 times per week) for more than 6 months in one’s life. History of iodized salt intaking was defined as having iodized salt for more than 6 months in one’s life. History of radiation exposure was defined as having radiation exposure (≥ 1 time per month) for more than 6 months in one’s life.

Difference of continuous variables was determined using independent sample *t* test, and difference of categorical variables was determined using chi-square test. If a *P* value was less than 0.05, it was considered to be statistically significant. Relationship between the history of IBD and the risk of PTC was determined using multivariate logistic regression analysis. Odds ratios (ORs), 95% confidence intervals (CIs), and *P* values were reported. Bonferroni correction was adopted for multiple analyses, and Bonferroni corrected *P* value was 0.004 (0.05/12 variables). If a *P* value was less than 0.004, it was statistically significant. These analyses were conducted using SPSS 17.0 (SPSS Inc., Chicago, IL, USA).

The study was separately approved by the ethics committee of Tianjin Medical University General Hospital (TMUGH-2016-0657) and Tianjin People’s Hospital (Tianjin Binjiang Hospital) (TJRMYY-2016-087).

### Meta-analysis

A structured literature search in MEDLINE/PubMed, EMBASE, Web of Science, and Cochrane Database of Systematic Reviews was conducted between January 1998 and December 2017. Search parameters were “Thyroid Cancer” [Title/Abstract], “Thyroid Neoplasms” [MeSH Terms], “Inflammatory Bowel Disease” [Title/Abstract], “Inflammatory Bowel Diseases” [MeSH Terms], “Ulcerative Colitis” [Title/Abstract], “Colitis, Ulcerative” [MeSH Terms], “Crohn’s Disease” [Title/Abstract], “Crohn Disease” [MeSH Terms] and their combinations.

Inclusion criteria were listed as follows: (1) There were IBD group and control group in each study. (2) Number of TC patients in each group was reported. Only English articles were included. Subsequently, references in the included articles were searched. If any reference met the inclusion criteria, it was also adopted.

Two trained reviewers independently performed the data selection according to a predefined survey form, which had several contents: first author, year of publication, country, study design, age at diagnosis, gender, period of follow-up, use of immunosuppressant, size of study, and so on. All data were double entered.

Our meta-analysis was conducted according to the recommendations from the Cochrane Collaboration and the Quality of Reporting of Meta-analyses guidelines [14, 15]. The fourfold table data (events/total in group one, events/total in group two) of each included study were input to a data matrix in Review Manager Version 5.2 (The Cochrane Collaboration, Software Update, Oxford). If *P* values for heterogeneity > 0.05, the results were performed by fixed effects method. If not, the random effects model was adopted. Then, OR and 95%CI were reported. It was statistically significant, if the 95% CI did not include the value one.

The study adopted three strategies to ensure the research quality. (1) Funnel plot obtained by Review Manager Software was used to evaluate the publication bias [16, 17]. (2) Sensitivity analysis was performed. In this process, each included study was sequentially removed from the meta-analysis to measure its contribution to the overall effect size. (3) All studies in the meta-analysis were scored by the Newcastle-Ottawa Scale to assess their overall quality [18].

## Results

### Case-control study

In Table 1, there were 1392 patients with IBD and 1392 controls with diverticulitis in the case-control study. Compared with the controls, body mass index was higher in the IBD patients (*P* < 0.001). More IBD patients had history of smoking, radiation exposure, or benign thyroid nodule (*P* < 0.001, *P* < 0.001, *P* < 0.001). More IBD patients had family history of TC (*P* < 0.001). Furthermore, the development of PTC was more common in the IBD patients than in the controls (*P* = 0.032).

**Table 1:** Characteristics of inflammatory bowel disease patients and controls in the case-control study

	IBD	Control	*P* value
Total (*n*)^b^	1392	1392	–
Male (*n*)	634	658	0.362
Age (years)^b^	37.0 ± 5.8	36.7 ± 5.8	0.454
BMI (kg/m^2^)^a^	23.2 ± 4.3	22.4 ± 4.3	< 0.001
History of smoking (*n*)	604	296	< 0.001
History of drinking (*n*)	410	395	0.531
History of iodized salt intaking (*n*)	754	708	0.081
History of radiation exposure (*n*)	251	14	< 0.001
History of benign thyroid nodule (*n*)	1064	570	< 0.001
Family history of TC (*n*)^a^	135	22	< 0.001
Age at diagnosis of IBD (years)^a^	26.9 ± 6.3	–	–
Duration of IBD on admission (years)	10.1 ± 2.8	–	–
Extraintestinal manifestations (*n*)	708	–	–
5-ASA treatment (*n*)^a^	1334	–	–
PTC development (*n*)^a^	11	3	0.032

^a^*BMI* Body mass index, *IBD* inflammatory bowel disease, *5-ASA* 5-aminosalicylic acid, *PTC* papillary thyroid carcinoma, *TC* thyroid cancer

^b^Categorical and continuous variables were separately showed by frequency and mean ± standard deviation

In Table 2, after adjusted for several confounding factors (i.e., age, gender, body mass index, smoking, drinking alcohol, iodized salt intaking, radiation exposure, benign thyroid nodule and family history of thyroid cancer), a multivariate logistic regression analysis reported that the history of smoking, radiation exposure, benign thyroid nodule, inflammatory bowel disease, or ulcerative colitis was associated with the increased risk of PTC (OR 3.81, 95%CI 1.27~11.37, *P* = 0.016; OR 5.37, 95%CI 1.78~16.13, *P* = 0.003; OR 9.22, 95%CI 1.21~70.54, *P* = 0.031; OR 3.70, 95%CI 1.04~13.26, *P* = 0.044; OR 4.12, 95%CI 1.12~15.24, *P* = 0.033). Family history of TC was also related to the elevated risk of the cancer (OR 13.02, 95%CI 4.47~37.97, *P* < 0.001). After Bonferroni correction, only history of radiation exposure and family history of TC reached the Bonferroni-corrected level of statistical significance.

**Table 2:** Association of several variables with risk of papillary thyroid carcinoma in the case-control study

Group	Subgroup	PTC^a^	Total	Univariate OR (95%CI)^a,b^	Univariate *P* value ^c^	Multivariate OR (95%CI)^b^	Multivariate *P* value^c^
Gender	Female	9	1492	Reference		Reference	
	Male	5	1292	0.64 (0.20~1.92)	0.425	0.65 (0.21~1.93)	0.424
Age (years)	< 36.9 years	6	1428	Reference		Reference	
	≥ 36.9 years	8	1356	1.41 (0.49~4.06)	0.529	1.42 (0.49~4.07)	0.528
BMI (kg/m^2^)^a^	< 22.8 kg/m^2^	5	1408	Reference		Reference	
	≥ 22.8 kg/m^2^	9	1376	1.85 (0.61~5.54)	0.272	1.86 (0.62~5.54)	0.271
History of smoking	Absent	5	1884	Reference		Reference	
	Present	9	900	3.80 (1.27~11.36)	0.017	3.81 (1.27~11.37)	0.016
History of drinking	Absent	7	1979	Reference		Reference	
	Present	7	805	2.47 (0.86~7.07)	0.092	2.48 (0.87~7.08)	0.092
History of ISI^a^	Absent	4	1322	Reference		Reference	
	Present	10	1462	2.26 (0.72~7.24)	0.167	2.27 (0.72~7.25)	0.166
History of RE^a^	Absent	9	2519	Reference		Reference	
	Present	5	265	5.36 (1.78~16.12)	0.003	5.37 (1.78~16.13)	0.003
History of BTN^a^	Absent	1	1150	Reference		Reference	
	Present	13	1634	9.21 (1.20~70.53)	0.032	9.22 (1.21~70.54)	0.031
Family history of TC^a^	Absent	8	2627	Reference		Reference	
	Present	6	157	13.01 (4.47~37.96)	< 0.001	13.02 (4.47~37.97)	< 0.001
History of IBD^a^	Control	3	1392	Reference		Reference	
	IBD	11	1392	3.69 (1.04~13.25)	0.045	3.70 (1.04~13.26)	0.044
History of CD^a^	Control	3	1392	Reference		Reference	
	CD	2	370	2.52 (0.43~15.12)	0.313	2.53 (0.43~15.13)	0.313
History of UC^a^	Control	3	1392	Reference		Reference	
	UC	9	1022	4.11 (1.12~15.23)	0.034	4.12 (1.12~15.24)	0.033

^a^*BMI* body mass index, *ISI* iodized salt intaking, *RE* radiation exposure, *BTN* benign thyroid nodule, *IBD* inflammatory bowel disease, *UC* ulcerative colitis, *CD* Crohn’s disease, *TC* thyroid cancer, *PTC* papillary thyroid carcinoma, *OR* odds ratio, *CI* confidence interval

^b^Univariate analysis was not adjusted by potential confounding factor. Multivariate analysis was adjusted by age, gender, body mass index, smoking, drinking alcohol, iodized salt intaking, radiation exposure, benign thyroid nodule, and family history of thyroid cancer

^c^Bonferroni correction was adopted for multiple analyses, and Bonferroni corrected *P* value was 0.004 (0.05/12 variables). If a *P* value was less than 0.004, it was statistically significant

### Meta-analysis

**Fig. 1:**
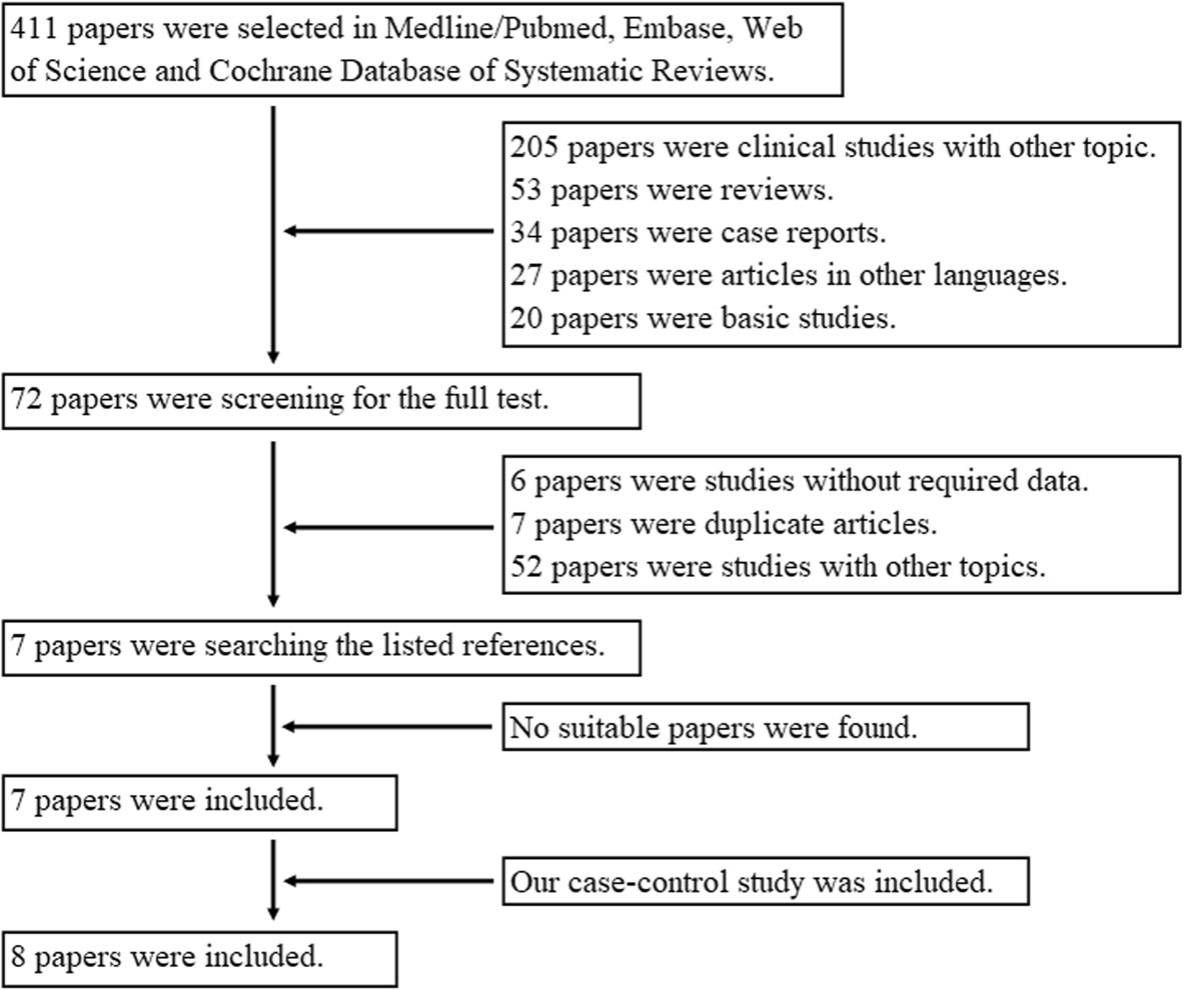
Paper checklist and flowchart in the literature search

As shown in Fig. 1, during the literature search using the parameters described above, 411 potential articles were found. Their abstracts were carefully reviewed, and 339 articles were directly excluded from the meta-analysis. Major reasons were that they were clinical studies with other topic (205), reviews (53), case reports (34), articles in other languages (27), or basic studies (20). Full texts of the remaining 72 articles were collected and reviewed. Among them, six articles focusing on this topic without required data, seven duplicate articles, and 52 articles focusing on other topics were excluded from the meta-analysis. Then, seven articles were included [11, 13, 19–23]. Their references were researched, and no suitable article was found. Meanwhile, our case-control study was also adopted. Thus, a total of eight studies were included in the meta-analysis.

Major characteristics of the included studies are presented in Table 3. There were 334,015 patients with IBD in the meta-analysis. The study with the largest sample size had 289,935 IBD patients, and the study with the smallest sample size had 374 patients. During the follow-up of more than 339,558 person-years, 244 patients had suffered from TC. Though time range of the literature search was 1998–2017, most of the included articles were published in the last 5 years. Improved detection for TC in the recent years was believed to be one of the possible reasons to explain this phenomenon. Five studies had normal controls, and the other three studies had patient controls with diverticulitis.

**Table 3:** Characteristics of included studies in the meta-analysis

First author	Year	Country	No. of IBD (*n*)^a^	IBD male (*n*)	IBD age at diagnosis (years)	IBD type	No. of controls (*n*)	Control type	Quality score
This study	2018	China	1392	634	Mean 26.9	Single center	1392	Patient	7
Jung	2017	Korea	15,291	9743	Mean 38.6	Population	1,529,100	Normal	7
So	2017	China	1603	893	Median CD 40/UC 53 ^a^	Population	160,300	Normal	7
Wadhwa	2016	USA	289,935	125,635	Mean 50.1	Population	315,145	Patient	7
Jussila	2013	Finland	21,964	11,810	NM	Population	2,196,400	Normal	7
Yano	2013	Japan	770	531	Mean 23.1	Single center	77,000	Normal	6
Sonu	2013	USA	2686	913	Mean 47.5	Single center	1638	Patient	6
Jess	2004	Denmark	374	157	NM	Population	37,400	Normal	7

^a^*IBD* inflammatory bowel disease, *NM* not mentioned, *CD* Crohn’s disease, *UC* ulcerative colitis

**Fig. 2:**
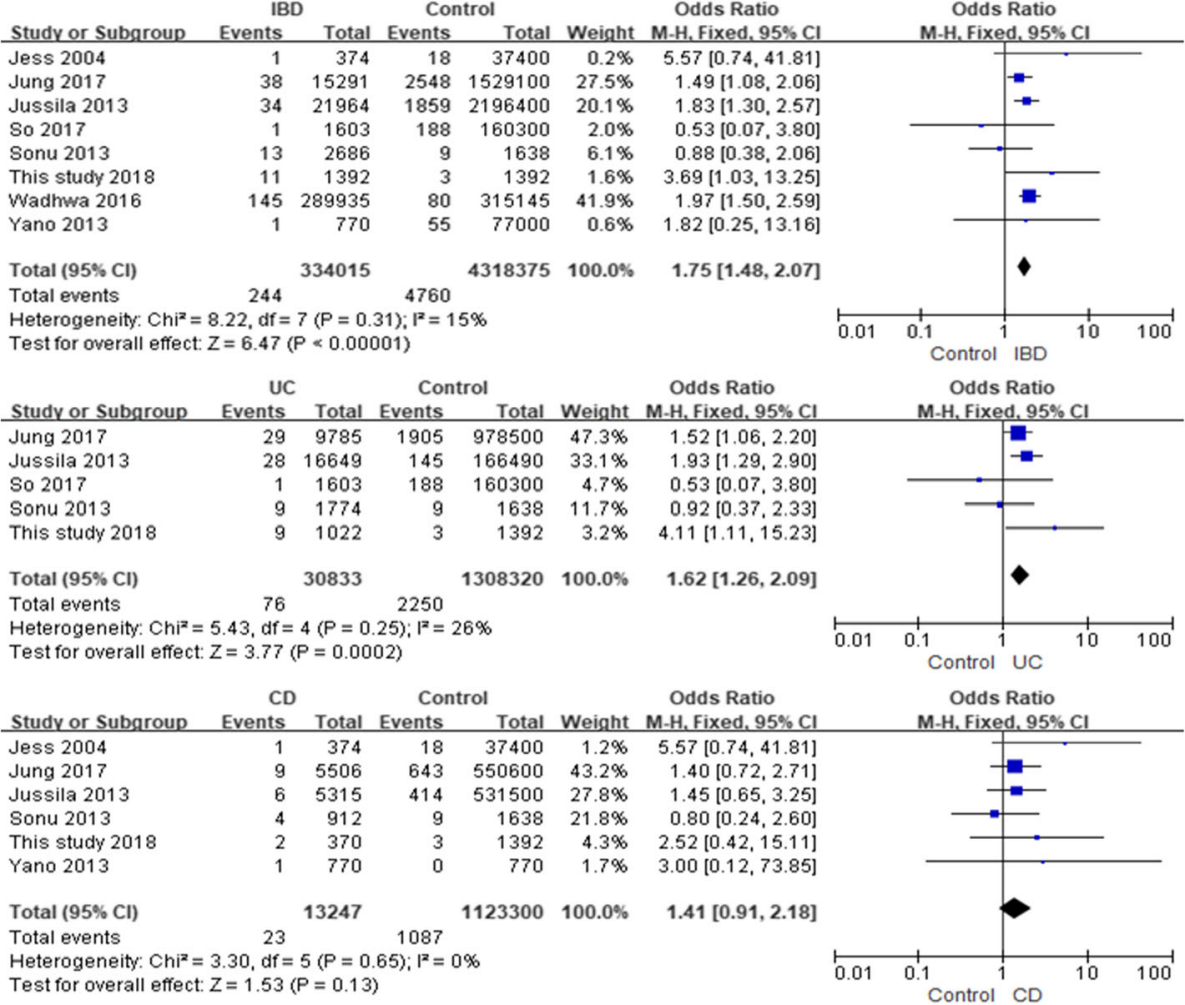
Relationship between history of inflammatory bowel disease and risk of thyroid cancer in fixed effects model

In Fig. 2, eight studies focused on the relationship between the history of IBD and the risk of TC in the patients. The fixed-effect pooled OR was 1.75 (95%CI 1.48~2.07, *P* for heterogeneity = 0.31). After excluding our study, the fixed-effect pooled OR was 1.72 (95%CI 1.45~2.04, *P* for heterogeneity = 0.33).

Five studies explored the possible association between the history of UC and the risk of TC, and the fixed-effect pooled OR was 1.62 (95%CI 1.26~2.09, *P* for heterogeneity = 0.25) (Fig. 2). After excluding our study, the fixed-effect pooled OR was 1.54 (95%CI 1.19~2.00, *P* for heterogeneity = 0.32).

Six studies showed the relationship between the history of CD and the risk of TC. The fixed-effect pooled OR was 1.41 (95%CI 0.91~2.18, *P* for heterogeneity = 0.65) (Fig. 2). After excluding our study, the fixed-effect pooled OR was 1.36 (95%CI 0.86~2.14, *P* for heterogeneity = 0.57).

In the subgroup analysis, the data were separately stratified by several factors, such as gender, race, case type, control type, use of immunosuppressant, and number of IBD patients. The results were showed in Table 4. The meta-analysis with hospital-based or small sample size (less than 10,000 cases) studies did not report any statistically significant results, revealing that study design had great influence on the result.

**Table 4:** Results of meta-analysis in the subgroup

Subgroup	No. of studies (*n*)	No. of IBD (*n*)^a^	OR (95%CI)^a^	*P* value for heterogeneity
Gender				
Male	2	21,553	2.05 (1.38~3.05)	0.26
Female	2	15,702	1.48 (1.11~1.97)	0.79
Race				
Asian	4	19,056	1.55 (1.15~2.08)	0.39
Caucasian	2	22,338	1.87 (1.34~2.61)	0.29
Case type				
Population	5	329,167	1.77 (1.49~2.11)	0.36
Hospital	3	4848	1.50 (0.78~2.88)	0.18
Control type				
Normal	5	40,002	1.61 (1.28~2.02)	0.48
Patient	3	292,621	1.89 (1.47~2.44)	0.12
Immunosuppressant				
Ever use	2	16,894	1.43 (1.04~1.96)	0.31
Never use	2	4078	1.47 (0.74~2.92)	0.07
No. of IBD patients				
≥ 10,000	3	327,190	1.79 (1.50~2.14)	0.42
< 10,000	5	5433	1.39 (0.78~2.48)	0.19

^a^*IBD* inflammatory bowel disease, *OR* odds ratio, *CI* confidence interval

Jung et al. and So et al. reported that 35.3% and 40.0% of the IBD patients separately in their studies received the immunosuppressant, and these two studies were defined as “ever use” studies [13, 19]. Sonu et al. in their study and our case-control study reported that the IBD patients did not adopt any kind of immunosuppressant before the development of TC, and these studies were defined as “never use” study [22]. Then, a subgroup analysis was done (Table 4). The IBD patients who ever received the immunosuppressant showed the increased the risk of TC, but the patients who never received these drugs did not show any change of the TC risk compared with the controls.

## Discussion

At present, only a few clinical and epidemiological studies focused on the risk of TC in IBD patients around the world. Most of the studies were based on their national health or medical insurance databases and had a huge sample size [11, 13, 19, 20, 23]. Because many potential confounding factors were not taken into account, these studies actually conducted an unadjusted analysis. In addition, there were two hospital-based studies. One was from Yano et al. and examined all kinds of cancers in CD patients [21]. Another one came from Sonu et al., and their results were only adjusted by age and gender [22]. Both the studies did not have a well-adjusted design. Therefore, we carried out this case-control study, which had taken into account many confounding factors, such as family history, radiation exposure, iodine intake abnormality, and obesity. We believed that our study might supplement the shortcomings of the previous studies.

Incidence of TC in the case-control study seemed to be higher than many previous studies [19, 20]. Possible reasons were listed as follows: First, TC was a relatively quiet tumor, and could be asymptomatic for a long time. Second, due to the improvement of health consciousness, many local people began to receive regular physical examination. Third, great progress had been made in diagnostic technology for TC. So, many cases of TC were found in recent years. The incidence of the cancer might be underestimated in the previous studies [19, 20].

Based on the adjusted analysis, the case-control study reported that the patients with IBD or its subtype UC had a more than 2-time or 3-time increased risk of PTC compared with the controls. However, these results did not reach the Bonferroni-corrected statistical significance.

In the meta-analysis, there were eight case-control studies with a total of 334,015 IBD patients. All the eligible studies had been included. Based on the pooled results, the patients with IBD showed a 45–100% increased risk of TC and the patients with its subtype UC showed a 20–100% increased risk of TC compared with the controls. The pooled results demonstrated the significant increase of the cancer risk in patients with IBD or its subtype UC. In the subgroup analysis, the relationship between the history of IBD and the risk of TC always existed in men, women, Asians, and Caucasians, suggesting that the increased risk of TC in IBD might not be a single race or gender problem.

To our knowledge, it was the first meta-analysis exploring the relationship between the history of IBD and the risk of TC. Pedersen et al. obtained an estimate of the risk of extra-intestinal cancer in CD and UC by performing a meta-analysis of population-based cohort studies and reported that the risk of upper gastrointestinal tract cancer, lung cancer, urinary bladder cancer, or skin cancer was significantly increased among patients with CD, and the risk of liver-biliary cancer or leukemia was significantly increased in patients with UC [9]. However, only two cohort studies with not enough subjects focused on TC, and their pooled results did not demonstrate a relationship between the history of IBD and the risk of TC [9]. Another meta-analysis from Huai et al. explored the impact of IBD on cholangiocarcinoma, and suggested the risk of cholangiocarcinoma was significantly increased among IBD patients, especially in intrahepatic cholangiocarcinoma cases [24]. Though there were no positive or available results on TC, both meta-analyses demonstrated the relationship between the history of IBD and the risk of extra-intestinal cancer.

In addition, the results of the case-control study were basically the same as those of the meta-analysis, though the Bonferroni-corrected results in the case-control study were not statistically significant. A possible explanation for the negative results after Bonferroni-correction was the lack of sample size. This explanation had been confirmed in the subgroup meta-analysis. The population-based studies in the subgroup meta-analysis reported a 50–110% increased risk of TC in the IBD patients. But, the studies with small sample size failed to do so.

At present, immunosuppressant became one common therapeutic drug for IBD. Some previous studies reported that the use of immunosuppressant increased the risk of cancer [25, 26]. In particular, immunosuppressant caused lymphoproliferative disorder, which was related to the development of TC [26]. In the case-control study, the patients did not receive the immunosuppressant and provided a negative result. In the meta-analysis, the patients who ever use immunosuppressant showed an increased risk of TC, but the patients who never used the drug did not. So, the strategy of immunosuppressive therapy might be a possible carcinogenic factor for TC.

Radiation exposure was another important confounding factor. A previous study reported that the IBD patients frequently received X-ray or computed tomography examination, which was a potential hazard to the human body [27]. In the case-control study, the history of radiation exposure was related to the increased risk of the cancer. Therefore, excessive exposure to radiation in IBD patients probably contributed to the development of TC.

Potential role of IBD in the development of TC could not be completely negated. The meta-analysis found that the risk of TC was higher in the UC patients than in the CD patients. The case-control study supported it, though the results did not reach Bonferroni-corrected statistical significance. This finding apparently could not be explained by the use of immunosuppressant and excessive exposure to radiation, but could be partly explained by chronic inflammation in IBD. It was well known that the pathogenesis of UC and CD was not exactly the same. CD was clearly identified as a Th1 inflammation [28], and Th2 mainly improved the development of UC [29]. Interleukin-13 (IL-13) was the key Th2 cytokine, and affected epithelial tight junctions, apoptosis and cell restitution in UC [30]. IL-5 was another Th2 cytokine, which could attract eosinophils to intestinal tissue and caused tissue damage and intestinal inflammation in UC [31]. Furthermore, Th2 cytokines (IL-4, IL-5 and IL-13), but not Th1 cytokines, were implicated in the pathogenesis of TC. Simonovic et al. suggested that peripheral blood cells of differentiated TC patients produced significantly higher concentrations of Th2 cytokines (IL-5 and IL-13) than control subjects, and radioactive 131-I therapy led to reduced secretion of these Th2 cytokines [32]. Joshi et al. demonstrated that IL-4 receptor-α (IL-4Rα) was overexpressed in anaplastic TC and might represent a novel therapeutic target in this cancer [33]. Taken together, there was no enough evidence that IBD was an independent risk factor for TC, but IBD might provide an appropriate inflammatory environment and promote the development of the cancer.

**Fig. 3:**
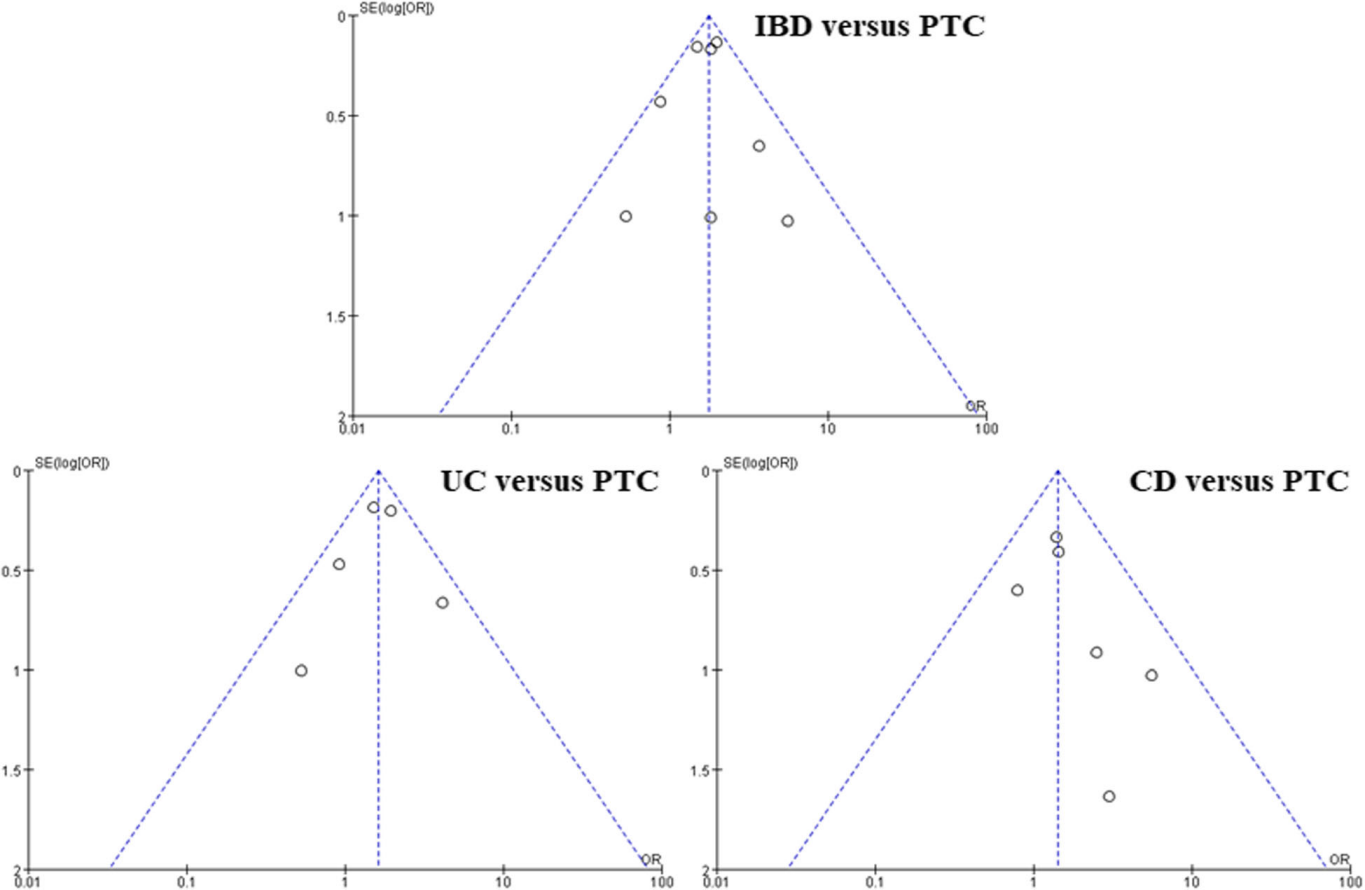
Funnel plots of studies evaluating publication bias in the meta-analysis

Quality evaluation of the meta-analysis was conducted and mainly involved three aspects: participant selection, group comparability, and outcome assessment. All included studies received satisfactory scores (Table 3). Major results were based on the fixed effects model (*P* values for heterogeneity were > 0.05). So, included studies showed good homogeneity. Publication bias was an important problem and could not be ignored. As shown in Fig. 3, the studies created roughly funnel-shaped distributions and formed symmetric graphs. So, significant publication bias had not been detected.

“Reverse causality” might be a potential limitation in case-control study. But, the studies in our meta-analysis were nested case-control studies with follow-ups of 6000–236,000 person-years. “Recall bias” was another potential problem in retrospective study. But, all included studies obtained their research data from medical records or health data system. The cases of IBD and TC were collected according to ICD-10 system. So, we did not think these issues affected our results.

TC was classified into medullary cancer, follicular cancer, undifferentiated cancer, and PTC. The case-control study focused on the PTC patients. But, most of the included studies in the meta-analysis explored the association of IBD with total TC. This limitation might cause bias, though PTC was the most common tissue subtype and accounted for more than 80% of total TC cases. More research should be conducted in different tissue types of TC.

## Conclusions

In conclusion, there were some defects in the current studies, but their achievements could be the basis for future research. Based on the results of our case-control study and meta-analysis, we suggested that the risk of TC probably elevated in patients with IBD, and this increase was more significant in its subtype UC. Inflammatory response of IBD itself, use of immunosuppressant and other undefined factors contributed to the development of TC. Well-adjusted and population-based studies should be conducted to verify our conclusion.

## Abbreviations

CD: Crohn’s disease
CI: Confidence interval
IBD: Inflammatory bowel disease
>OR: Odds ratio
PTC: Papillary thyroid carcinoma
TC: Thyroid cancer
UC: Ulcerative colitis
